# Outcome of intraoperative brachytherapy as a salvage treatment for locally recurrent rectal cancer

**DOI:** 10.1007/s00066-024-02271-1

**Published:** 2024-08-08

**Authors:** Raluca Stoian, Hannes P. Neeff, Mark Gainey, Michael Kollefrath, Simon Kirste, Constantinos Zamboglou, Jan Philipp Harald Exner, Dimos Baltas, Stefan Fichtner Feigl, Anca-Ligia Grosu, Tanja Sprave

**Affiliations:** 1 Department of Radiation Oncology, University Hospital of Freiburg, Robert-Koch-Straße 3, 79106 Freiburg, Germany; 2 German Cancer Consortium (DKTK) Partner Site Freiburg, German Cancer Research Center (dkfz), Neuenheimer Feld 280, 69120 Heidelberg, Germany; 3 Faculty of Medicine, University of Freiburg, 79106 Freiburg, Germany; 4 Department of General and Visceral Surgery, Medical Center—University of Freiburg, Faculty of Medicine, University of Freiburg, Hugstetter Straße 55, 79106 Freiburg, Germany; 5 German Oncology Center, University Hospital of the European University Cyprus, Limassol, Cyprus

**Keywords:** Rectal cancer, Recurrence, Boost, Intraoperative radiotherapy, High-dose radiotherapy

## Abstract

**Background:**

Locally advanced recurrent rectal cancer (RRC) requires a multimodal approach. Intraoperative high-dose-rate brachytherapy (HDR-BT) may reduce the risk of local recurrence. However, the optimal therapeutic regimen remains unclear. The aim of this retrospective monocentric study was to evaluate the toxicity of HDR-BT after resection of RRC.

**Methods:**

Between 2018 and 2022, 17 patients with RRC received resection and HDR-BT. HDR-BT was delivered alone or as an anticipated boost with a median dose of 13 Gy (range 10–13 Gy) using an ^192^iridium microSelectron HDR remote afterloader (Elekta AB, Stockholm, Sweden). All participants were followed for assessment of acute and late adverse events using the Common Terminology Criteria for Adverse Events version 5.0 and the modified Late Effects in Normal Tissues criteria (subjective, objective, management, and analytic; LENT-SOMA) at 3‑ to 6‑month intervals.

**Results:**

A total of 17 patients were treated by HDR-BT with median dose of 13 Gy (range 10–13 Gy). Most patients (47%) had an RRC tumor stage of cT3‑4 N0. At the time of RRC diagnosis, 7 patients (41.2%) had visceral metastases (hepatic, pulmonary, or peritoneal) in the sense of oligometastatic disease. The median interval between primary tumor resection and diagnosis of RRC was 17 months (range 1–65 months). In addition to HDR-BT, 2 patients received long-course chemoradiotherapy (CRT; up to 50.4 Gy in 1.8-Gy fractions) and 2 patients received short-course CRT up to 36 Gy in 2‑Gy fractions. For concomitant CRT, all patients received 5‑fluorouracil (5-FU) or capecitabine. Median follow-up was 13 months (range 1–54). The most common acute grade 1–2 toxicities were pain in 7 patients (41.2%), wound healing disorder in 3 patients (17.6%), and lymphedema in 2 patients (11.8%). Chronic toxicities were similar: grade 1–2 pain in 7 patients (41.2%), wound healing disorder in 3 patients (17.6%), and incontinence in 2 patients (11.8%). No patient experienced a grade ≥3 event.

**Conclusion:**

Reirradiation using HDR-BT is well tolerated with low toxicity. An individualized multimodality approach using HDR-BT in the oligometastatic setting should be evaluated in prospective multi-institutional studies.

## Introduction

Despite optimized and personalized multimodality treatment of primary rectal cancer, recurrent rectal cancer (RRC) remains a challenge.

Locally advanced rectal cancer (LARC; T3–4 or N +) is generally treated with a combination of surgery, radiotherapy, and chemotherapy [1]. The standard treatment for LARC is neoadjuvant chemoradiotherapy (CRT). Neoadjuvant CRT achieves superior local control with a more favorable toxicity profile as compared to adjuvant CRT [2–6]. Local recurrence rates after neoadjuvant (C)RT and total mesorectal excision (TME) are 4–7% [2, 4, 5]. However, the excellent result of this standard therapy is fraught with acute and long-term side effects [7, 8]. Therefore, many concepts are currently being evaluated for de-escalation and toxicity reduction on the one hand and for improving the response on the other. The addition of induction chemotherapy before neoadjuvant CRT prolongs disease-free survival without improving locoregional control [2]. Long-term results in patients with LARC after neoadjuvant therapy alone with complete or near-complete tumor regression justify the omission of TME [9, 10]. Remarkably, neoadjuvant chemotherapy alone with selective CRT for a poor response is comparable to neoadjuvant CRT in LARC in terms of local control and disease-free survival [11].

**Fig. 1:**
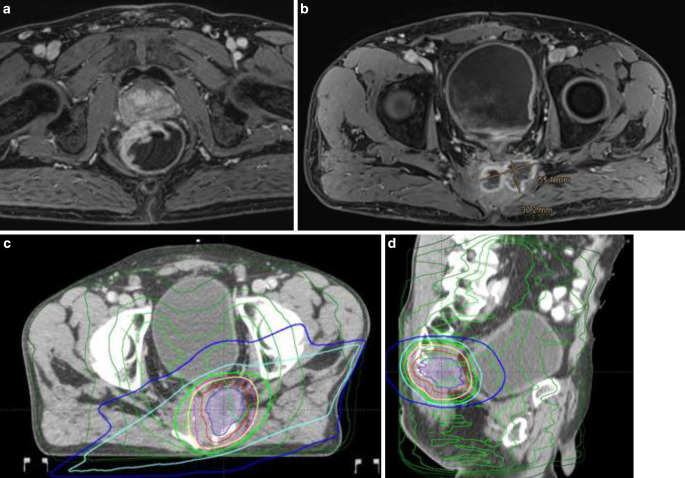
Case presentation. A 52-year-old man was initially diagnosed with locally advanced moderately differentiated adenocarcinoma of the rectum mrT4a, N2b, EMVI+ in June 2018 (**a**). This was followed in July–September 2018 by neoadjuvant chemoradiotherapy (according to the Sauer protocol: radiotherapy total dose 50.4 Gy, single dose 1.8 Gy; chemotherapy 1000 mg/m^2^ KOF 5‑fluorouracil weeks 1 and 5) and, following a good response, ultra-deep anterior rectal resection in October 2018; postoperative tumor classification: ypT2 ypN0 (0/12) L0 V0 Pn0 R0. March 2019: initial diagnosis of unilateral pulmonary metastasis in the sense of oligometastasis, which was treated with an atypical resection. February 2020 and March 2021: unilateral pulmonary oligoprogression, which was again treated by atypical resection. In November 2021, a presacral local recurrence of the previous rectal cancer was confirmed (MRI, contrast-enhanced T1w). **b** In December 2021, a second course of neoadjuvant chemoradiotherapy of the presacral local recurrence was performed up to 36 Gy total dose in 1.8-Gy single doses with capecitabine. **c**, **d** *Green lines* show the reconstructed isodoses from the first radiotherapy as part of the neoadjuvant chemoradiotherapy after rigid coregistration. The isodoses from the second CRT are shown as follows: *yellow* 95%, *green* 80%, *light blue* 50%, and *dark blue* 20% of the applied dose. Macroscopic tumor (gross tumor volume) in *violet*, clinical target volume (CTV) in* red*, planning volume (PTV) in *purple*. December 2021: salvage resection with high-dose-rate brachytherapy (1 fraction of 10 Gy) was performed using a 4 × 6 cm Freiburg flap, with positive resection margins detected perioperatively but not in the final histological results rpT3, rpN0 (0/13), L0 V0 Pn1 R0

However, there is no consensus regarding the optimal management of RRC after standard multimodal neoadjuvant therapy [12, 13]. If possible, complete radical resection—as the optimal curative option for RRC—should be the aim [14]. A second neoadjuvant re-irradiation (re-RT) with or without (induction) chemotherapy can achieve tumor regression and enable R0 resection ([15–17]; Fig. 1). Most patients with RRC have been pretreated with pre- or postoperative radiotherapy with a dose of approximately 50 Gy over 5 weeks or 25 Gy in 1 week [18, 19]. Therefore, the dose of percutaneous neoadjuvant or adjuvant re-RT is limited due to the severe toxicity of the rectum and the surrounding organs [20]. Nevertheless, neoadjuvant normofractionated re-(C)RT up to 30 Gy can achieve results comparable to the first course of neoadjuvant CRT [21]. Moreover, local dose escalation can be performed using high-dose-rate intraoperative RT (HDR-BT) with simultaneous protection of organs at risk [22]. HDR-BT could improve local recurrence-free survival, especially for patients with resection with microscopic residuals (R1), which substantially increases the risk of a new recurrence [23, 24]. Radical RRC surgery after neoadjuvant CRT and simultaneous metastasectomy in the presence of visceral oligometastatic disease may be another option in this selected cohort [25].

## Materials and methods

The aim of this retrospective study was to evaluate the toxicity of the personalized approach consisting of HDR-BT and resection of RRC. Patients treated with HDR-BT during surgery alone or as tumor bed boost with external-beam radiation therapy (EBRT) from 2018 to 2022 at the University Hospital Freiburg were retrospectively included in this study. Institutional criteria for selecting patients at a high risk of recurrence for HDR-BT alone or as an anticipated boost included patients with potentially resectable locally RRC, debulking surgery, close or positive margins, or oligometastatic disease.

Patients diagnosed with locally RRC were discussed and evaluated by a specialized tumor board. All recurrences were confirmed by biopsy. Before a decision on multimodal treatment was made, preoperative restaging by pelvic MRI and CT of the thorax and abdomen was performed. Systemic therapy was performed according to current guidelines and recommendations of the interdisciplinary oncology panel.

Intraoperative radiotherapy (IORT) was performed using an ^192^iridium microSelectron HDR remote afterloader (Elekta AB, Stockholm, Sweden) in a shielded operating room. Due to the complex anatomical surfaces, the Freiburg flap applicator (Nucletron, Veenendaal, the Netherlands) was used in individually tailored sizes for each case. The flexible Freiburg flap consists of interconnected silicon spheres with a diameter of 1 cm. Thus, the effective distance from the source in the radiation guide tube to the applicator surface is 5 mm. The prescription dose (range 10–13 Gy) was applied to the depth 5 mm from the applicator surface.

Neoadjuvant or adjuvant EBRT was applied using conventional fractionation (36–50.4 Gy in 18–28 fractions). CT-based (Brilliance, CT Big Bore, Philips, Cleveland, OH, USA) three-dimensional treatment planning (Oncentra MasterPlan; Nucletron, Veenendaal, the Netherlands and/or the Eclipse™ planning system; Varian Medical Systems, Palo Alto, CA, USA) was performed using tangential portals (6 or 18 MV; Synergy; Elekta, Crawley, UK). Intensity-modulated RT (IMRT) or volumetric modulated arc therapy (VMAT) was used to reduce the bowel and bladder dose. The EBRT was performed using surface-guided RT (C-RAD Catalyst; C‑RAD AB, Uppsala, Sweden). The target volume included the recurrent tumor with a safety margin. Concomitant with EBRT, capecitabine 825 mg/m^2^ was administered twice daily. In the neoadjuvant approach, restaging was performed 4–6 weeks after completion of chemoradiotherapy (CRT). Subsequently, at 8–10 weeks after CRT, resection and HDR-BT were completed.

All patients were monitored by a surgeon and a radiation oncologist every 3 to 6 months for the first 2 years, followed by annual visits thereafter. Acute postoperative side effects (up to 90 days) were evaluated according to the Common Terminology Criteria for Adverse Events version 5.0 (CTCAE v.5). Late toxicity was judged using the modified Late Effects in Normal Tissues criteria (subjective, objective, management, and analytic; LENT-SOMA).

### Statistical analysis

Outcomes were defined from the date of IORT during surgery for locally recurrent rectal cancer to the pertinent event. Dates are reported as a mean, median (range), and frequencies. Statistics were performed with SPSS version 29 (IBM, Armonk, NY, USA).

## Results

A total of 17 patients treated by HDR-IORT with a median of 13 Gy (range 10–13 Gy) were identified and included in this analysis. Demographic characteristics at the time of recurrence and those related to the nature of RCC are summarized in Table 1. All patients had histologically confirmed locally RRC, most frequently presacral or anterior, with 4 cases each (23.5%; Table 1). Most patients (47%) had an RRC tumor stage of cT3–4 N0. In 13 cases (76.4%) the RRC was located within fewer than 5 cm of the anocutaneous line. Median age was 60 years (range 39–84) in the mainly female collective 10 (58.8%; Table 1). The median interval between primary tumor resection and diagnosis of RRC was 17 months (range 1–65 months).

**Table 1:** Patient and tumor characteristics of patients treated by high-dose-rate brachytherapy in our institution between 2018 and 2023 (*n* = 17). Staging of recurrent rectal cancer was based on the 8th edition of the UICC TNM classification

	*n* (%)
*Total patients*	17
*Sex*	
Male	7 (41.2)
Female	10 (58.8)
Age, years (median)	60
*Tumor stage of recurrence*	
cT3‑4 N0	8 (47)
cT3–4 N +	4 (23.6)
cTx N0	5 (29.4)
*Distant metastases of recurrence*	
cM0	10 (58.8)
cM1a	1 (5.9)
cM1b	2 (11.8)
cM1c	4 (23.5)
*Distance from anocutaneous line*	
<5 cm	13 (76.4)
5–10 cm	2 (11.8)
>10 cm	2 (11.8)
*Localization of recurrence*	
Lateral	3 (17.6)
Presacral	4 (23.5)
Anterior	4 (23.5)
Anastomosis	1 (5.8)
Perineum	2 (11.7)
Other	3 (17.6)

*cTx* tumor could not be assessed, *M* metastases

In addition to HDR-BT, 2 patients received long-course CRT up to 50.4 Gy in 1.8-Gy fractions and 2 other patients received short-course CRT up to 36 Gy in 2‑Gy fractions (Table 2). For concomitant CRT, all patients received 5‑fluorouracil (5-FU) or capecitabine. The use of long-course CRT for RRC was only in RT-naïve patients. Short-course re-CRT was performed in pre-irradiated patients. Chemotherapy before RRC resection was prescribed in 10 patients (58.8%) but was not completed by any (Table 2).

**Table 2:** Treatment characteristics of the rectal cancer primary diagnosis and the local recurrence (*n* = 17)

	Treatment of primary tumor	Treatment of recurrence
	*n* (%)	*n* (%)
*Total patients*	17	17
*CRT*		
*Long-course CRT*		
50.0/50.4 Gy	9 (52.9)	2 (11.8)
45 Gy	1 (5.9)	–
*Shot-course CRT*		
25 Gy without chemotherapy	1 (5.9)	–
36 Gy with chemotherapy	–	2 (11.8)
Prescribed chemotherapy	13 (76.5)	10 (58.8)
Completed chemotherapy	12 (70.6)	–
Completed radiotherapy	10 (58.8)	4 (23.5)
*cCR*		
Yes	1 (5.9)	4 (23.5)
Partial	7 (41.2)	2 (11.8)
No	9 (52.9)	3 (17.6)
Tumor growth	–	8 (47.1)
*Type of resection*		
APR	5 (29.4)	3 (17.6)
LAR	6 (35.3)	1 (5.9)
Hartmann	2 (11.8)	–
Other	4 (23.6)	13 (76.5)
*Continence-preserving surgery*		
Yes	9 (52.9)	4 (23.5)
No	8 (47.1)	13 (76.5)
*Resection status*		
R0	6 (35.3)	5 (29.4)
R1	2 (11.8)	4 (23.5)
R2	2 (11.8)	1 (5.9)
RX	7 (41.2)	3 (17.6)
Irresectable	–	4 (23.5)
*pCR*		
Yes	7 (41.2)	3 (17.6)
No	10 (58.8)	14 (82.4)
*Histology*		
Adenocarcinoma	16 (94.1)	13 (76.5)
Mucinous	–	–
Other	1 (5.9)	4 (23.5)
*Grading*		
G1	1 (5.9)	–
G2	14 (82.4)	6 (35.3)
G3	2 (11.8)	2 (11.8)
G4	–	–
Unspecified	–	9 (52.9)

*APR* abdominoperitoneal resection, *cCR* clinical complete response, *CRT* chemoradiotherapy, *LAR* low anterior resection, *pCR* pathological complete response, *R* resection status

Preoperative MRI detected a complete response (cCR) in only 4 patients (23.5%) and a partial response (cPR) in 2 patients (11.8%; Table 2). Finally, a pathological (p)CR was detected in more than half of the lesions (*n* = 9, 52.9%; Table 2).

For surgical resection of RRC, the most common method was an individual approach in 13 (76.5%) and abdominoperineal resection (APR) in 3 patients (17.6%; Table 2). Perioperatively, after initial RRC resection and before HDR-BT, evaluation of frozen sections showed irresectable tumors in 4 patients (23.5%) and an R1 resection in the remaining patients. However, the perioperative R1 resection status was revised in the final histopathologic reprocessing. In the final histopathological analysis, an R0 situation was detected in 5 (29.4%), R1 in 4 (23.5%), R2 in 1 (5.9%), and, finally, RX in 3 patients (17.6%; Table 2). Adenocarcinoma was most common, with 13 cases (76.5%) in the resected RRC specimens. Undifferentiated carcinoma was found in 9 (52.9%) and grade 2 in 6 cases (35.3%) in the final histology (Table 2).

Continence-preserving surgery for RRC was possible in only 4 patients (23.5%), the remaining 13 (76.5%) received a definitive stoma (Table 2).

Median follow-up was 13 months (range 1–54). At the time of RRC diagnosis, 7 patients (41.2%) had visceral metastases (hepatic, pulmonary, or peritoneal) in the sense of oligometastatic disease (Table 1). All patients were treated with HDR-BT with curative intent.

Table 3 displays the toxicity profile of the study population. No patient experienced a higher than grade ≥3 event. Acute and chronic toxicities had grade 1 to 2 manifestations. The most common acute toxicity grade 1–2 was pain in 7 patients (41.2%), wound healing disorder in 3 (17.6%), and lymphedema in 2 patients (11.8%; Table 3). Chronic toxicities were similar: pain in 7 patients (41.2%), wound healing disorder in 3(17.6%), and incontinence in 2 patients (11.8%; Table 3).

**Table 3:** Acute and chronic radiotherapy-related toxicities after high-dose-rate brachytherapy according to the Common Terminology Criteria for Adverse Events (CTCAE v5.0) and the modified Late Effects in Normal Tissues criteria (subjective, objective, management, and analytic; LENT-SOMA)

	*n* (%)
*Total patients*	17
*Cumulative toxicity grade 1–2*	
Any acute toxicity reported	10 (58.8)
Acute radiodermatitis	2 (11.8)
Acute pain	7 (41.2)
Acute incontinence	1 (5.9)
Acute diarrhea	1 (5.9)
Acute wound healing disorder	3 (17.6)
Acute lymphedema	2 (11.8)
Any chronical toxicity reported	8 (47.1)
Chronic radiodermatitis	1 (5.9)
Chronic pain	7 (41.2)
Chronic incontinence	2 (11.8)
Chronic diarrhea	1 (5.9)
Chronic wound healing disorder	3 (17.6)
Chronic lymphedema	1 (5.9)

Due to the short follow-up and small size of the cohort, the survival curves were not included in the analysis.

## Discussion

Over the past decade, immense interdisciplinary developments in the multimodal treatment of primary rectal cancer have resulted in a further reduction of local recurrence rates. The contemporary RRC collective was predominantly pretreated with standard therapy using TNT with TME, which renders renewed local curative therapy using re-CRT and surgery significantly more difficult.

The resection status at the time of RRC surgery is considered a decisive prognostic factor for the development of local (re-)recurrence [24]. In the case of initial inoperability in non-irradiated RRCs, conversion to complete resectability can be achieved in over 60% with dose-escalated neoadjuvant CRT [26]. Therefore, the positive resection margins imply an indication to perform HDR-BT [24, 27]. Percutaneous neo- or adjuvant dose escalation during irradiation can possibly achieve an additional improvement in the local control of RRC [15, 28, 29]. In view of the unclear evidence, we performed this single-institutional retrospective study to evaluate the toxicity of multimodal salvage treatment using HDR-BT and surgery in RRC patients.

Historical RRC cohorts after HDR-BT (range 10–20 Gy) showed 2‑ and 5‑year local control rates of 63–72% and 39%, respectively [24, 30, 31]. Positive resection margins were confirmed as the most significant predictor for LRFS, OS, and disease-free survival. Interestingly, Sorrentino et al. found that neoadjuvant re-CRT significantly improves disease-free survival for both negative and especially R1 resection margins [32]. However, after neoadjuvant re-CRT and surgery, G3 mucositis was observed in 33.3% and G4 toxicity in 15.2% of patients: 4 rectal perforations and rectovaginal or rectovesical fistulae [32].

Alektier et al. demonstrated 2‑year LRFS, OS, and DMFS rates after HDR-BT and surgery of 55%, 75%, and 67%, respectively [30]. The authors found the most common toxicity to be wound healing complications (24%) and damage to the ureter (23%), bladder (20%), and peripheral nerves (16%). Alektier et al. also emphasized the difficulty in clearly separating surgery- and radiation-related toxicities or sequelae of RRC itself [30]. In our group, comparable acute and late complications of wound healing disorders were observed, with 17.6% each (Table 3).

Interestingly, Voogt et al., in a technique comparison of HDR-BT vs. IOERT, found superior LRFS in the R1 situation in favor of HDR-BT [23]. This significant improvement in local control in the HDR-BT cohort compared to IOERT was associated with a significantly higher rate of serious complications, with 46% and 26% (*p* = 0.017), respectively [23]. Thus, Voogt et al. recorded the following most common major complications: presacral abscess (26%), urinary tract leakage (12%), and abdominal wall wound dehiscence (8%). In our cohort, a considerably higher wound healing disorder rate was observed (Table 3). The authors elucidated the HDR-BT benefit over IOERT and formulated the following multifactorial hypothesis: a major advantage of HDR-BT is the ability to irradiate a concave extended surface [23] in areas that are anatomically difficult to access. This is an essential limitation of IOERT, because the rigid applicators are poorly suited to curved areas or narrow spaces. Another potential advantage of better local control in favor of HDR-BT is a much higher dose at the surface: about 150–170% of the prescribed dose at a depth of 10 mm, whereas IOERT delivers a homogeneous dose at the surface of the target area, which is the equivalent of the prescribed treatment dose [23]. This significant increase in the prescribed dose directly at the surface during HDR-BT can lead to local necrosis and may explain the increased complication rate.

Re-CRT with moderate doses of 30 to 40 Gy to the localized target volume has mild toxicity [15]. In the prospective study by Valentini et al. after neoadjuvant hyperfractionated re-CRT with 59 study participants, only 7 patients experienced late toxicities, 2 of which were skin fibrosis, 2 impotence, 2 urinary tract complications, and 1 small bowel fistula [15]. In our cohort, the cumulative late toxicity rate was numerically higher at 8 (47.1%), with chronic pain reported most frequently (in 7 patients, 41.2%; Table 3). The historical cohort in Mohiuddin et al. using a conventional three-dimensional irradiation technique showed some grade 4 adverse acute toxicity events (6/103, 6%) and only grade 3 chronic toxicity [33]. Mohiuddin et al. identified the single daily fraction size and an interval of less than 24 months before re-RT as significant factors influencing late toxicity [33]. In contrast, in our study, no higher-grade toxicities were observed after the median follow-up of 13 months. On the one hand, in our study the follow-up is too short to detect chronic toxicity and on the other, implementation of the image-guided intensity-modulated technique led to better protection of the surrounding organs [12]. However, dose-escalated percutaneous re-RT with a median of 50 Gy (median cumulative total dose in 2-Gy fractions [EQD2] with an α/β ratio of 3: 105.84 Gy) after previous pelvic pre-irradiation causes significant grade 4 late toxicities such as fistula in 1 (2.4%) and bowel obstruction in 2 patients (4.9%) [34]. Röder et al. reported a complication rate of 59% after intraoperative RT by electron beam IOERT (10–20 Gy) with or without external (C)RT (median dose 41.4 Gy), in which postoperative wound healing disorders and abscess or fistula formation were recorded at 20% and 16%, respectively [29]. These results are comparable to our cumulative acute adverse event rates of 58.8% (Table 3).

Due to the limited oncological benefit, curative-intent local therapy of RRC has so far been preferred for non-metastatic disease [13, 15, 16, 31, 35]. The current population-based cohort study by Swartjes et al. from the Netherlands recorded a 3-year RRC rate of approximately 6%, with synchronous distant metastases in 44.9% (3-year rate of 3%) [13]. The incidence of distant metastasis is comparable to our detected rate of 41.2% visceral oligometastasis in our RRC cohort. It is conceivable that simultaneous resection of the RRC after neoadjuvant CRT and all oligometastases can achieve a further oncological benefit [25].

The following agents for optimization of treatment in LARC, which will contribute to a substantial therapeutic change in the RRC collective, should be mentioned: additional use of galunisertib but not of pembrolizumab in neoadjuvant CRT shows increased rates of pathological response [36, 37]. Promising short-term results have been shown in LARC with mismatch repair deficiency using mono-agent dostarlimab and with subsequent avoidance of CRT and TME in the case of clinical complete response [38]. If RT cannot be avoided in the TNT approach for LARC, the appropriate choice of treatment for RRC remains a challenge.

Despite these promising results, the limitations of this analysis should be noted. This retrospective study was conducted in one institution only and included a heterogeneous cohort with individual treatment concepts and various HDR-BT doses. Patients were carefully selected for HDR-BT based solely on intraoperatively positive frozen section margins. This may have led to overtreatment in 5 patients (29.4%) with an R0 resection situation under consideration of the final histologic results (Table 3). Furthermore, only 4 patients were irradiated percutaneously, with different doses. Therefore, in our small cohort of 4 patients, the true benefit of additional percutaneous dose saturation in terms of improved local control cannot be conclusively assessed, which limits the transferability to other patient groups outside our institution. The short follow-up in our cohort provides limited evidence on late toxicities and oncological survival benefit that may occur over a longer period.

In summary, re-irradiation with HDR-BT is well tolerated and has a mild toxicity profile. An individualized multimodal approach using HDR-BT for oligometastatic disease should be evaluated in prospective multi-institutional studies.

## Data Availability

The data used in this analysis are available with the authors’ permission.

## Abbreviations

CR: Complete response
CRT: Chemoradiotherapy
HDR-BT: High-dose-rate intraoperative brachytherapy
IOERT: Intraoperative radiotherapy using electrons
IORT: Intraoperative radiotherapy
LARC: Locally advanced rectal cancer
LRFS: Local recurrence-free survival
OS: Overall survival
PR: Partial response
RRC: Recurrent rectal cancer
RT: Radiotherapy
TME: Total mesorectal excision
VMAT: Volumetric modulated arc therapy
